# Pattern of progression of intrahepatic cholangiocarcinoma: Implications for second‐line clinical trials

**DOI:** 10.1111/liv.15117

**Published:** 2021-12-10

**Authors:** Francesco Tovoli, Ingrid Garajová, Fabio Gelsomino, Massimo Iavarone, Piera Federico, Massimiliano Salati, Matilde Corianò, Francesco Caputo, Stefania De Lorenzo, Alessandro Granito, Matteo Renzulli, Bruno Daniele, Fabio Piscaglia

**Affiliations:** ^1^ Division of Internal Medicine, Hepatobiliary and Immunoallergic Diseases IRCCS Azienda Ospedaliero‐Universitaria di Bologna Bologna Italy; ^2^ Medical Oncology Unit University Hospital of Parma Parma Italy; ^3^ Department of Oncology and Hematology University Hospital of Modena Modena Italy; ^4^ Foundation IRCCS Ca' Granda Ospedale Maggiore Policlinico Division of Gastroenterology and Hepatology Milan Italy; ^5^ U.O.C. Oncologia Ospedale del Mare Napoli Italy; ^6^ Oncology Unit Azienda USL Bologna Bologna Italy; ^7^ Department of Medical and Surgical Sciences University of Bologna Bologna Italy; ^8^ Department of Radiology IRCCS Azienda Ospedaliero‐Universitaria di Bologna Bologna Italy

**Keywords:** biliary tract cancer, cholangiocarcinoma, liver cancer, outcome, prognosis

## Abstract

**Background:**

Intrahepatic cholangiocarcinoma (iCCA) is the second most frequent liver cancer. The overall survival of iCCA and other biliary tract cancers (BTC) remains poor. Recently, the ABC‐06 trial reported the superiority of FOLFOX vs clinical observation as a second‐line treatment. Still, the survival benefit was less than expected. We hypothesized that the pattern of progression of iCCA can drive post‐progression survival (PPS), similar to hepatocellular carcinoma.

**Methods:**

Multicentre retrospective evaluation of consecutive iCCA patients who progressed after frontline systemic treatment with gemcitabine as monotherapy or in combination with platinum. Radiological assessment of progression was evaluated according to RECIST 1.1. The progression pattern was divided according to the presence/absence of new extrahepatic lesions (NEH).

**Results:**

We included 206 patients from 5 centres. The median OS was 14.1 months and its independent predictors (hazard ratio [HR], 95% confidence interval [CI]) were previous surgery 0.699 [0.509‐0.961], performance status >2.445 [1.788‐3.344], permanent first‐line discontinuation 16.072 [5.102‐50.633], registration of ascites 2.226 [1.448‐3.420] or bilirubin >3 mg/dl 3.004 [1.935‐4.664] during the follow‐up, and disease progression 2.523 [1.261‐5.050]. The appearance of NEH independently predicted OS 2.18 [1.55‐3.06] in patients with radiological progression. Amongst 138 patients eligible for second‐line treatment, PPS was 16.8 and 5.9 months in cases without and with NEH, respectively (*P* = .001). Progression owing to NEH lesions was an independent predictor of PPS 1.873 [1.333‐2.662], together with performance status, time to progression to the frontline treatment, bilirubin >3 mg/dl and ascites.

**Conclusions:**

PPS of iCCA is influenced by progression pattern, with important implications for second‐line trial design and analysis.

**Abbreviations**
BTC: biliary tract cancer
GEM: gemcitabine
GEMCIS: gemcitabine‐cisplatin
GEMOX: gemcitabine‐oxaliplatin
HCC: hepatocellular carcinoma
iCCA: intrahepatic cholangiocarcinoma
NASH: non‐alcoholic steatohepatitis
NEH: new extrahepatic lesion
OS: overall survival
PPS: post‐progression survival
TTP: time to progression

**Key points**

- Pattern of progression influences post‐progression survival of various cancers.
- The role of pattern of progression for intrahepatic cholangiocarcinoma is unknown.
- Appearance of new extrahepatic lesions (NEH) was related to worse outcomes.
- At progression, metastatic patients had different outcomes according to NEH.
- Pattern of progression of iCCA has repercussions for clinical trials.

## 1 INTRODUCTION

Intrahepatic cholangiocarcinoma (iCCA) is the second most frequent primary liver cancer, following hepatocellular carcinoma (HCC).^1^ Similar to HCC, iCCA can be diagnosed in patients with pre‐existing chronic liver disease during surveillance.^2^ Currently, iCCA is responsible for 20% of liver‐related deaths.^3^ For this reason, iCCA attracts the interest of both oncologists and hepatologists. Moreover, the current epidemic of non‐alcoholic steatohepatitis is causing a rise in the cases of both HCC and iCCA, increasing the proportion of iCCA patients amongst biliary tract cancers (BTC) and amplifying the scientific interest towards this once rare neoplasm.^4^, ^5^, ^6^ Both HCC and iCCA had little to no effective systemic treatments until 10‐15 years ago, with sorafenib and gemcitabine‐cisplatin (GEMCIS) proving superiority to placebo and gemcitabine monotherapy (GEM) in 2007 and 2010, respectively.^7^, ^8^ While a second line for HCC was identified in 2017^9^ and further treatments proved effective in most recent years, the search for second‐line treatment for iCCA is still not concluded.^10^

Recently, the ABC‐06 randomized controlled trial (RCT) demonstrated the advantage of modified FOLFOX over clinical observation in overall survival (OS) in a population of 162 BTC patients, in which iCCA was the predominant tumour type.^11^ However, the OS improvement was less than expected (6.2 vs 5.3 months), owing to surprisingly high survival in the clinical observation group. While the reasons behind this survival remain elusive, iCCA patients still have unmet needs.

The search for prognostic tools for iCCA is a hot topic.^12^, ^13^ The determinants of post‐progression survival (PPS) after a frontline therapy for iCCA have been scantly investigated.^14^ The pattern of radiological progression has been shown to be an important driver of PPS in HCC progressing to sorafenib^15^, ^16^ and in extrahepatic malignancies.^17^, ^18^, ^19^ In the case of HCC, the pattern of progression is now recognized as one of the critical elements in post‐sorafenib trials and has been explicitly investigated in RCTs of second‐line agents.^20^, ^21^ We hypothesized that the pattern of progression might also have a role in determining the PPS of iCCA, with implications in understanding some findings of the ABC‐06 trial, optimizing the design of future second‐line trials and informing patients in clinical practice.

## 2 METHODS

This retrospective study considered all patients with iCCA evaluated in five centres between January 2012 and December 2018 for first‐line systemic treatment because of unresectable disease.

### 2.1 Outcomes and assessments

OS and time to progression (TTP) were calculated from the start of the frontline systemic treatment to death and disease progression, respectively. Radiological evaluation of response was done by computed tomography (CT) scan according to the response evaluation criteria in solid tumours (RECIST) v.1.1.^22^ We categorized the type of progression as proposed by Reig et al.^15^ Briefly, all patients with the appearance of new metastatic lesions were classified as progressors owing to new extrahepatic lesions (NEH). The remaining patients with radiological progression (ie ≥20% increase in tumour size against a known baseline lesion and/or new intrahepatic lesion) were considered to have a pattern of progression without NEH.

Radiology assessment was blinded to the evolution and outcome. Patients who died before the first imaging assessment were classified as progressors. PPS was measured from the date of detecting progression at radiology until the date of death or last follow‐up.

The predictors of OS were determined in the whole cohort. We also assessed the impact of progression pattern on OS and PPS in patients with radiological progression and performed subanalyses of patients who had preserved PS and liver function at the time of progression and were therefore a candidate to second‐line treatment. Given the heterogeneity of the enrolment criteria of second‐line iCCA and BTC trials, we simulated three scenarios at the progression: (1) patients with PS 0/1, bilirubin <3 mg/dl, no clinically relevant ascites, first‐line treatment with any gemcitabine‐containing regimen (similar to the pemigatinib and ivosidenib trials^23^, ^24^); (2) same characteristics but limited to patients who received gemcitabine in combination with any platinum agent (simulating the regorafenib REACHIN trial and the bintrafusp alfa INTR@PID BTC 047 study)^25^, ^26^; (3) same clinical characteristics but restricted to patients who received GEMCIS as a frontline treatment (similarly to the ABC‐06 trial).^11^

In the latter scenario, we also verified the role of sensitivity to platinum, as it was recognized as a prognostic factor after progression to GEMCIS in the ABC‐06 trial.^11^ Platinum sensitivity was defined as ‘sensitive’ (progression after 90 days of day 1 of the last cycle of frontline GEMCIS), resistant (progression within the first 90 days after completion of day 1 of the last cycle of frontline GEMCIS) or refractory (progression during frontline GEMCIS)^11^

Finally, we verified whether the information about the pattern of progression could refine the prognostic ability of the Association des Gastro‐Entérologues Oncologues (AGEO) CT2BIL score.^14^

### 2.2 Treatments

GEMCIS and GEMOX (gemcitabine‐oxaliplatin) doublets were prescribed as the standard of care. Similar to other European centres, GEMOX was used as an equivalent to GEMCIS, owing to better tolerance and simpler outpatient administration. Management of toxicities included gemcitabine and platinum dose reductions or permanent discontinuation. GEM monotherapy was prescribed only in patients who had a contraindication to platinum at the baseline or who permanently discontinued platinum.

### 2.3 Follow‐up evaluations

Clinical and laboratory assessments were done before each chemotherapy administration. Radiology tumour evaluation was usually performed first at week 8 and afterwards approximately every 12 weeks.

### 2.4 Statistical analysis

Categorical variables are described as frequencies and percentages and continuous variables as median and the interquartile range (IQR). Times to event data were estimated by Kaplan‐Meier curves. Fisher's exact test was used to compare categorical variables. The Mann‐Whitney method was used to compare ordinal and continuous variables.

To define the predictors of OS, we took into account the following baseline parameters: sex, age, performance status [PS (0/1/2)], largest tumour diameter (mm), multinodular disease (yes/no), nodal involvement (yes/no), distant metastasis (yes/no), total bilirubin (mg/dl), ascites (yes/no) and carbohydrate antigen 19‐9 (Ca19‐9) (IU/ml). Moreover, we also assessed the impact of registering the following categorical variables (yes/no): total bilirubin ≥3 mg/dl, the appearance of palpable ascites, dose reduction, permanent discontinuation of systemic drugs, deterioration of PS (using PS 0 as a reference) and changes in CA19‐9 (>37 IU/ml). All statistics involving evolutionary events were done by time‐dependent covariate analyses.^15^ Cox univariate and multivariate regression models with time‐dependent covariates were used to estimate hazard ratios (HR). Statistical analyses were performed using SPSS version 24.0 and Stata version 16.0.

### 2.5 Ethics

The Ethics Committee approved the study (protocol 78/2017/O/OSSN), which was conducted according to the 1975 Declaration of Helsinki. Considering the retrospective design and the unfavourable prognosis of the investigated disease, the Ethics Committee waived the need for informed consent for deceased patients and for patients whose clinical conditions had worsened to a point in which they were not able to sign a valid consent. All of the remaining patients provided written informed consent for this study.

## 3 RESULTS

Amongst 332 iCCA patients included in the database of the participating centres in the timeframe of the study, 241 had received first‐line systemic treatment. Amongst this population, 206 patients were enrolled and 35 excluded as they did not receive gemcitabine‐containing regimens. At the time of the database lock (February 2021), the median follow‐up was 12.2 months (range 0.4‐82.3); 172 patients died and 2 were still receiving first‐line treatment.

### 3.1 Baseline parameters

Demographic, clinic and laboratory characteristics are summarized in Table 1. Seventy‐eight patients (37.8%) had received prior surgery, and 30 (14.6%) had previously received adjuvant systemic treatment.

**TABLE 1:** Characteristics of the study population (n = 206) at the start of the first‐line systemic treatment. Categorical variable are expressed as median (interquartile range). Continuous variables are reported as frequencies (percentage)

Variable	
Age (years)	63 (42‐81)
Male sex	137 (66.5)
Liver cirrhosis	44 (21.4)
Previous surgery	78 (37.8)
Previous biliary drainage	22 (10.7)
Performance status	
0	115 (55.8)
1	67 (32.5)
2	24 (11.7)
Main tumour size (mm)	56 (30‐86)
Multinodular disease	139 (67.5)
N1	104 (50.5)
M1	91 (44.2)
Bilirubin (mg/dl)	0.84 (0.61‐1.42)
Ca19‐9 (IU/l)	284 (67‐871)
First‐line regimen	
GEMCIS	84 (40.8)
GEMOX	76 (36.9)
GEMCTABINE	46 (22.3)

Abbreviations

GEMCIS, gemcitabine‐cisplatin; GEMOX, gemcitabine‐oxaliplatin.

### 3.2 Treatment

First‐line treatment regimens included: GEMCIS (40.8%), GEMOX (36.9%) and GEM (22.3%). The median duration of treatment was 4.0 months (95% CI 3.5‐4.4). Most patients (72.8%) required at least one dose modification. Amongst the 160 patients who received a platinum‐containing doublet, 35.6% discontinued platinum permanently for toxicities. Permanent discontinuation of gemcitabine occurred in 7 patients (3.4%). Consequently, progressive disease was the leading cause of permanent interruption of the first‐line therapies (96.6%).

Amongst the remaining evolutionary events, bilirubin >3 mg/dl occurred in 18 (8.7%) patients and palpable ascites was documented in 10 (4.9%) cases. The most frequent causes of hyperbilirubinemia were biliary obstruction (n = 10), followed by liver failure (n = 6), and cholangitis (n = 2). Imaging evidence of peritoneal carcinomatosis and/or positive cytology of the peritoneal fluid was found in the majority (n = 7) of ascitic patients.

### 3.3 Radiological response

The best radiological response was progressive disease, stable disease and partial response in 51.0, 42.2 and 6.8% of patients, respectively. No complete responses were reported. Tumour progression occurred in all but four patients (98.1%). Median TTP, PFS and OS were 5.1 (95% CI 4.0‐6.2), 4.3 (95% CI 3.7‐5.0) and 14.1 months (95% CI 11.4‐16.9), respectively. The median OS in patients with tumour progression with and without NEH lesions was 22.4 and 9.8 months, respectively (*P* < .001).

### 3.4 Predictors of OS

The univariate analysis of the whole population identified the following 5 baseline predictors of survival: previous surgery, multinodular disease, nodal involvement, ECOG‐PS and GEM monotherapy (Table S1). The multivariable Cox regression restricted them to ECOG‐PS and prior surgery (Table 2).

**TABLE 2:** Multivariable Cox analysis of overall survival

Variables	Whole study cohort (n = 206)			Radiological tumour progression (n = 178)			Candidates for second‐line trials (n = 138)		
	HR	95% CI	*P*	HR	95% CI	*P*	HR	95% CI	*P*
Baseline									
Previous surgery	0.699	0.509‐0.961	.027	0.700	0.501‐0.978	.049	0.720	0.473‐1.032	.069
Performance status >0	2.445	1.788‐3.344	<.001	2.173	1.545‐3.056	<.001	1.894	1.263‐2.840	.002
Main tumour size (mm)									
Multinodular disease	1.344	0.970.1.863	.076	1.299	0.920‐1.834	.137	1.352	0.844‐2.165	.209
N1	1.223	0.892‐1.676	.211	1.138	0.810‐1.599	.456	1.096	0.788‐1.733	.439
Ca19‐9 (IU/l)	1.006	0.981‐1.030	.478	1.004	0.978‐1.032	.521	1.005	0.980‐1.045	.211
Regimen									
GEMCIS		Reference			Reference			Reference	
GEMOX	0.971	0.679‐1.389	.871	1.180	0.802‐1.736	.400	1.186	0.715‐1.970	.509
GEMCITABINE	1.306	0.857‐1.990	.215	1.619	0.921‐2.569	.082	1.288	0.695‐2.387	.421
Evolutionary events									
Gemcitabine dose reduction									
First‐line permanent interruption	16.072	5.102‐50.633	<.001	25.011	7.818‐80.015	<.001	34.830	4.811‐252.165	<.001
Platinum withdrawal^a^	1.173	0.861‐1.596	.312	1.323	0.903‐1.752	.099	1.192	0.818‐1.738	.361
Ascites	2.226	1.448‐3.420	<.001	2.731	1.762‐4.234	<.001	3.203	1.839‐5.304	<.001
Bilirubin >3 mg/dl	3.004	1.935.4.664	<.001	3.414	2.148‐5.426	<.001	2.775	1.548‐4.976	.001
Tumour response									
Progression	2.523	1.261‐5.050	.009		All progressors			All progressors	
New extrahepatic lesion	‐	‐	‐	1.878	1.350‐2.612	<.001	1.882	1.292‐2.742	.001

Abbreviations

CI, confidence interval; GEMCIS, gemcitabine‐cisplatin; GEMOX, gemcitabine‐oxaliplatin; HR, hazard ratio.

^a^ For patients who received gemcitabine monotherapy, the time to event was set to 0 days.

Afterwards, we analyzed whether changes in the evolutionary covariates during the treatment had any impact on OS, with the statistical methodology that properly takes into account both baseline and evolutionary parameters.^15^, ^27^ The time‐dependent analysis identified 4 additional possible predictors of survival: registration of hyperbilirubinemia and ascites, permanent first‐line discontinuation, radiological progression. Because of GEM monotherapy, having resulted related to survival in the previous univariable analysis, we also included permanent platinum discontinuation (considering the time to event = 0 days in monotherapy patients) in a multivariable model. The multivariable Cox analysis, confirmed registration of ascites or bilirubin >3 mg/dl, definitive first‐line discontinuation and radiological tumour progression.

The baseline and evolutionary predictors were maintained after the exclusion of the 28 patients without radiological tumour progression. Moreover, progression due to new extrahepatic lesions was found to be an independent OS predictor in patients with radiological tumour progression (HR 1.878, 95% CI 1.350‐2.612, *P* < .001).

### 3.5 Post‐progression survival

The post‐progression analyses included 178 patients. Twenty‐eight patients were excluded as they died before the first imaging follow‐up (n = 24) or because they had not progressed at the time of the database lock (n = 4). The median PPS in patients with radiological progression was 9.0 months (95% CI 6.7‐11.3). Ascites, hyperbilirubinaemia and ECOG‐PS at the time of progression, together with TTP and progression owing to new extrahepatic lesions, were independent predictors of OS. (Table 3).

**TABLE 3:** Multivariate Cox analysis of post‐progression survival in patients with radiological tumour progression under a first‐line treatment for intrahepatic cholangiocarcinoma. Patients were considered eligible for second‐line trials at tumour progression under 3 scenarios: (a) performance status 0‐1, total bilirubin <3 mg/dl, no clinically relevant ascites, any gemcitabine‐containing first‐line treatment; (b) same as previous but allowing only patients who received gemcitabine‐platinum first‐line treatment; (c) same as previous but limited to patients who received gemcitabine‐cisplatin as first‐line treatment

Variable	Radiological tumour progression (N = 178)			Candidates for second‐line trials: Scenario A (*N* = 138)			Candidates for second‐line trials: Scenario B (*N* = 116)			Candidates for second‐line trials: Scenario C (N = 53)		
	HR	95% CI	*P*	HR	95% CI	*P*	HR	95% CI	*P*	HR	95% CI	*P*
PS>0	2.588	2.014‐3.327	<.001	2.062	1.364‐3.118	.001	2.446	1.522‐3.931	<.001	2.780	1.373‐5.627	.004
TTP (months)	0.958	0.930‐0.987	.005	0.954	0.925‐0.983	.002	0.954	0.924‐0.986	.005	0.956	0.916‐0.998	.001
NEH lesion	1.873	1.333‐2.632	<.001	2.174	1.475‐3.202	<.001	1.991	1.301‐3.045	.001	2.179	1.124‐4.224	.021
Bilirubin >3 mg/dl	2.824	1.333‐5.980	.007		Not applicable			Not applicable			Not applicable	
Ascites	2.506	1.320‐4.755	.005		Not applicable			Not applicable			Not applicable	

Abbreviations

CI, confidence interval; HR, hazard ration; NEH, new extrahepatic; PS, performance status; TTP, time to progression (referred to first‐line treatment).

### 3.6 Post‐progression survival in potential candidates for second‐line trials

**FIGURE 1:**
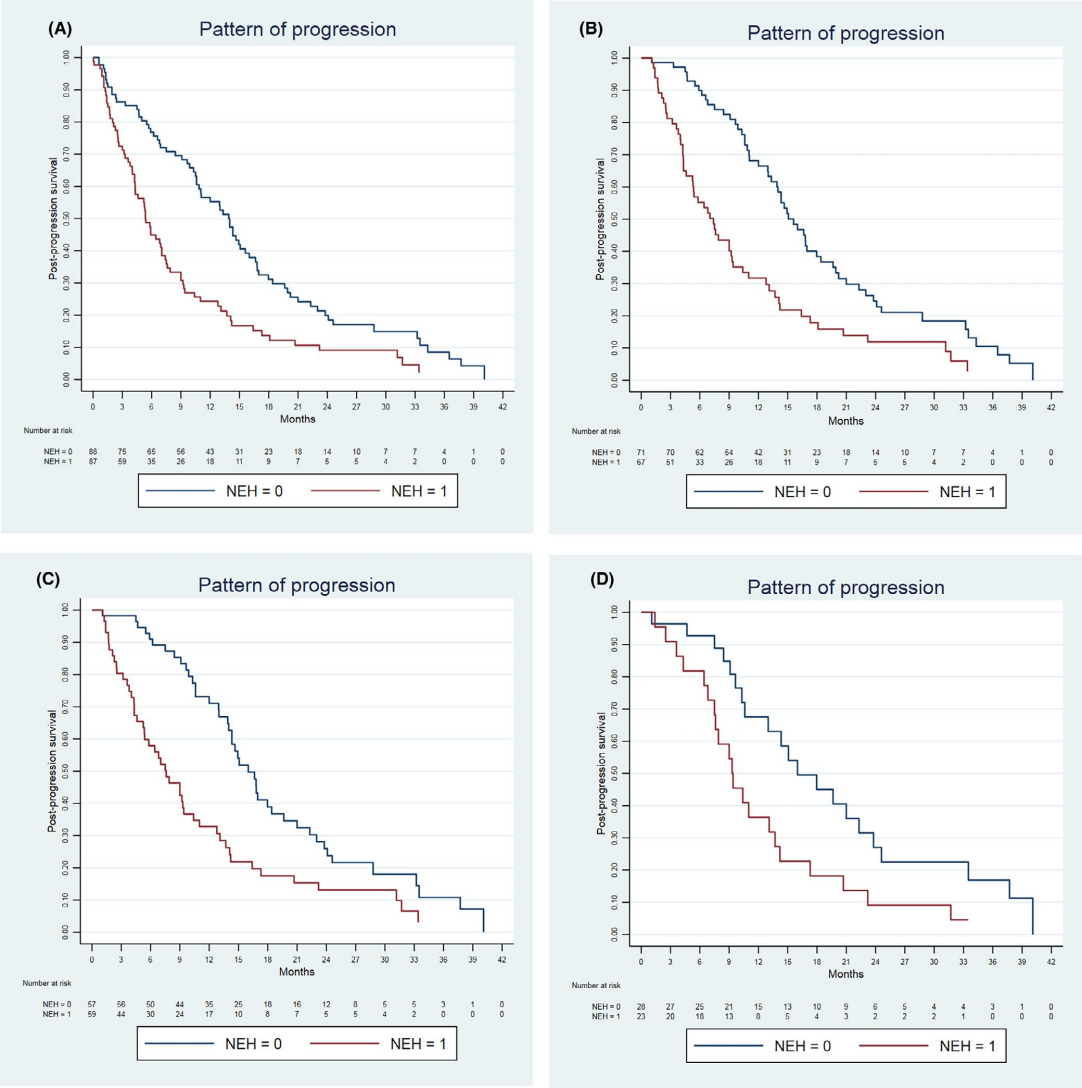
Post‐progression survival stratified according to the pattern of progression in patients with radiological tumour progression (A) and eligible for second‐line trials under 3 different scenarios: (B) performance status 0–1, total bilirubin <3 mg/dl, no clinically relevant ascites, any gemcitabine‐containing first‐line treatment; (C) same as previous but allowing only patients who received gemcitabine‐platinum first‐line treatment; (D) same as previous but limited to patients who received gemcitabine‐cisplatin as a first‐line treatment

**FIGURE 2:**
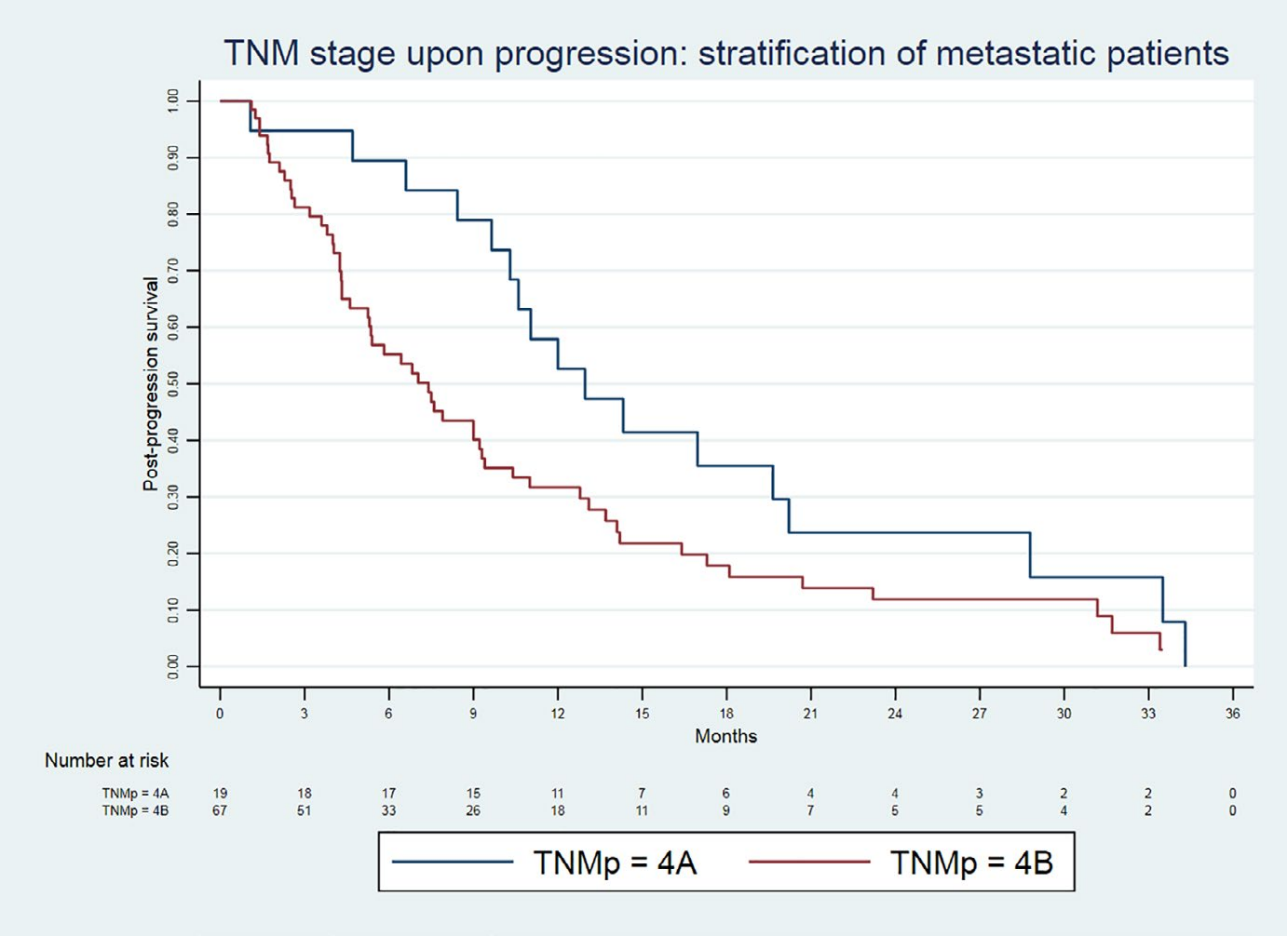
Post‐progression survival curves estimated from the Cox model in TNM Stage 4 patient candidates to second‐line trial divided according to the absence or presence of new extrahepatic lesions (n = 86). TNMp‐4A: Patients TNM Stage 4 under first‐line treatment with progression owing to the growth of existing nodules or new intrahepatic sites. TNMp‐4B: Patients TNM Stage 4 under first‐line treatment with progression owing to new extrahepatic lesions

We simulated different scenarios reflecting the enrolment criteria of the iCCA/BTC clinical trials (Table 3). The number of candidates for second‐line trials decreased according to the restrictiveness of the criteria (from 138 to 53 cases). In all scenarios, the PPS was significantly different according to the TTP, ECOG‐PS at progression and progression pattern. On the contrary, PPS was not related to the second‐line treatment (evaluated as FOLFOX‐4 vs others). The HR from a progression pattern characterized by NEH ranged from 1.991 (95% CI 1.301‐3.045, *P* < .001) to 2.179 (95% CI 1.124‐4.224, *P* = .021). The PPS survival curves stratified according to the progression pattern are depicted in Figure 1. Amongst patients who were metastatic upon progression, patients with NEH had a significantly worse PPS than those without it (7.5 vs 14.3 months, *P* = .027) (Figure 2).

A further multivariable model was necessary to assess the role of sensitivity to platinum, as this variable was tightly correlated and co‐linear with TTP. Amongst the 53 patients who progressed to GEMCIS and were eligible for second‐line therapy, 20 (37.7%) were sensitive to platinum, 22 (41.5%) refractory and 11 (20.8%) resistant. In the multivariable model, both platinum sensitivity (sensitive vs refractory/resistant) and pattern of progression were independent predictors of PPS (Table S2).

### 3.7 Refinement of the AGEO‐CT2BIL prognostic score

**FIGURE 3:**
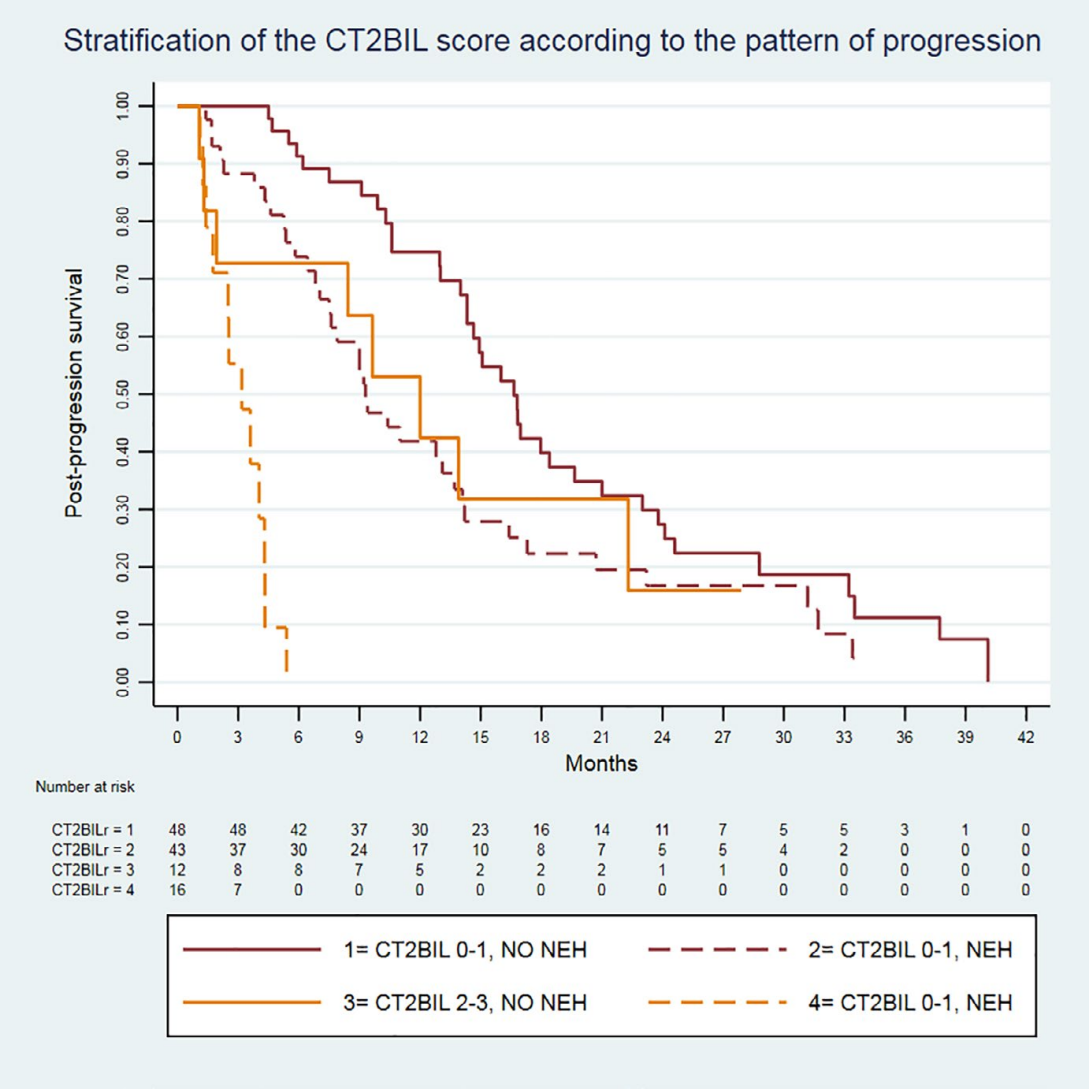
Survival curves of the Association des Gastro‐Entérologues Oncologues (AGEO) CT2BIL score amongst the 119 patients who received second‐line treatment, stratified according to the pattern of radiological progression under first‐line treatment

This analysis was performed in patients progressing to a gemcitabine‐platinum doublet who underwent a second‐line treatment. This population included all patients of scenario B, plus three patients who had an ECOG‐PS 2 at progression and received second‐line treatment, for a total of 119 patients. The CT2BIL score was as follows: 0 in 30 (25.2%) patients, 1 in 62 (52.1%) patients, 2 in 26 (21.8%) patients and 3 in a single patient (0.8%). Because of our sample size, the CT2BIL scores were categorized as low (0 or 1) or high (2 or 3). In a multivariable model, including CT2BIL score (low/high) and pattern of progression (NEH lesion yes/no), both variables independently predicted PPS (high CT2BIL: HR 3.743, 95% CI 2.019‐5.975, *P* < .001; NEH lesions: HR 2.555, 95% CI 1.653‐3.949, *P* < .001). PPS stratified according to the pattern of progression was 16.6 vs 9.3 months in patients with CT2BIL 0‐1 and 12.0 vs 3.6 months in cases of CT2BIL 2‐3 (Figure 3).

## 4 DISCUSSION

We demonstrated that progression correlated with survival, but also that specific patterns of progression implied different PPS and OS.

The simple and yet meaningful hypothesis that a new small intrahepatic progression does not have the same impact as the extrahepatic spread was known for HCC.^15^ No similar studies had been performed for iCCA. Still, this simple concept has deep implications in clinical practice and in the design of clinical trials. In particular, an imbalance in the distribution of the patients across the study arms may cause relevant effects, including trial failure. This issue is well known in HCC. A relative excess of patients with macrovascular invasion in the brivanib arm owing to the stratification of the patients according to a combined criterion of ‘extrahepatic spread and/or macrovascular invasion’ concurred to the failure of Phase 3 BRISK trial.^28^, ^29^ Similarly, it has been hypothesized that lenvatinib might have shown superiority to sorafenib (rather than a non‐inferiority) in Phase 3 REFLECT trial if the rate of patients with high alpha‐fetoprotein had been balanced in the 2 groups.^30^, ^31^ In the case of iCCA and BTC, Neuzeillet et al^14^ tried to address the lack of knowledge about second‐line survival in a large collaborative study. They found that previous surgery on the primary tumour, progression‐free survival >6 months, the reason for gemcitabine discontinuation, performance status and peritoneal carcinomatosis were prognostic factors. The authors also proposed and validated a simplified score (CT2BIL) based on all of the previous elements minus progression‐free survival.^14^ In comparison with Neuzillet et al,^14^ our study investigated the role of the progression pattern and explored other evolutionary events according to an established methodology.^15^ While adding novel information, we also validated most results provided by Neuzillet et al In particular, performance status was confirmed as a relevant PPS determinant in patients fit for a second‐line therapy together with TTP (surrogating PFS) and ascites (basically reflecting the presence of peritoneal carcinomatosis rather than liver failure). Also, we demonstrated that the implementation of the pattern of progression might refine the prognostic information provided by the CT2BIL score.

More importantly, we demonstrated that the pattern of progression independently predicted the PPS in patients eligible for clinical trials, regardless of the enrolment criteria. Even patients who were metastatic at progression had different PPS according to their pattern of progression (demonstrating that the prognostic value of pattern of progression does not merely reflect information from the stage). The authors of the ABC‐06 study did not specify whether the pattern of progression was considered as a possible confounding factor, so it is impossible to verify the actual impact that our finding had in this specific trial (and, in particular, whether an imbalance between the study groups occurred).^11^

Despite the strength of a relatively large population (about of the same size as that of the ABC‐06 trial) and of the analysis of time‐dependent events, our study also has some limitations. Firstly, this is a retrospective analysis: even if the patients were consecutively enrolled and clinical data were available for all of them, minor biases cannot be fully excluded. Secondly, our patients were heterogeneous in terms of the first and second‐line received. This aspect reflects the real‐world clinical practice; possible biases were excluded after checking that the pattern of progression was not related to the type of second‐line treatment received. Thirdly, we considered only patients with iCCA and thus our conclusions cannot be outright extended to the remaining BTC types. There are, however, some considerations in this regard. For instance, iCCA patients represented the largest portion of patients enrolled in the latest BTC trials and thus their outcomes can deeply influence the final results of BTC trials. Finally, iCCA displays some specific pathogenic alterations distinguishing it from other BTC, therefore, suggesting clinical trials specifically dedicated iCCA.

In conclusion, we found that the iCCA radiological pattern of progression influences the post‐progression outcomes, both in a pure population of progressors and in candidates to second‐line trials. This information can refine the information deriving from existing prognostic tools and have repercussions on the design of future clinical trials.

## CONFLICT OF INTERESTS

The authors declare that the following financial interests/personal relationships may be considered as potential competing interests: FT: consultant for Bayer and Eisai, the advisory board for Guerbet, lecture fees from Ipsen; MI: speaking and teaching for Bayer, Gilead Science, Janssen, BTG, AbbVie and consultant for BCG; AG: consultant for Bayer; BD: honoraria from Ipsen, AstraZeneca, Incyte, Lilly, Eisai, Bayer, Roche and MSD; FP: consultant for AstraZeneca, Bayer AG, EISAI, GE and Tiziana life sciences; speaker's bureau honoraria from Bayer AG, Bracco, EISAI and Laforce and research contract with Esaote; IG, FG, PF, MS, MC, FC, SD, MR: no conflicts to declare.

## ETHICS APPROVAL

The Ethics Committee approved the study (protocol 78/2017/O/OSSN), which was conducted according to the 1975 Declaration of Helsinki.

## TRIAL REGISTRATION NUMBER

Not applicable.

## PATIENT CONSENT STATEMENT

Considering the retrospective design and the unfavourable prognosis of the investigated disease, the Ethics Committee waived the need for informed consent for deceased patients and for patients whose clinical conditions had worsened to a point in which they were not able to sign a valid consent. All of the remaining patients provided written informed consent for this study.

## PERMISSION TO REPRODUCE MATERIAL FROM OTHER SOURCES

No material has been reproduced from other sources.

## Supporting information

Table S1

Table S2

## Data Availability

The data that support the findings of this study are available on request from the corresponding author. The data are not publicly available because of privacy or ethical restrictions.
