# Supplementary material for: Pattern of progression of intrahepatic cholangiocarcinoma: Implications for second‐line clinical trials
Source: Liver Int. 2021 Dec 10;42(2):458–67. doi: 10.1111/liv.15117 (PMC9300150; doi:10.1111/liv.15117)
Supplement: Supplementary file 1 — Table S1 [file LIV-42-458-s001.docx]

**SUPPLEMENTARY TABLE:** Univariate Cox analysis of overall survival

| **Variables** | Whole study cohort (n=206) | | |
| --- | --- | --- | --- |
|  | HR | 95% CI | P |
| **Baseline** |  |  |  |
| Age (years) | 1.001 | 0.974-1.028 | 0.978 |
| Male sex | 1.111 | 0.870-2.140 | 0.313 |
| Liver cirrhosis | 0.961 | 0.660-1.390 | 0.918 |
| Previous surgery | 0.672 | 0.490-0.922 | 0.014 |
| Previous biliary drainage | 1.299 | 0.937-1.854 | 0.112 |
| Performance status >0 | 2.423 | 1.766-3.307 | <0.001 |
| Main tumour size (mm) | 1.003 | 0.999-1..007 | 0.140 |
| Multinodular disease | 1.352 | 0.976-1.872 | 0.069 |
| N1 | 1.364 | 1.006-1.850 | 0.046 |
| M1 | 1.159 | 0.856-1.569 | 0.339 |
| Ca19-9 (IU/l) | 1.005 | 1.002-1.009 | 0.041 |
| Regimen |  |  |  |
| - GEMCIS |  | Reference |  |
| - GEMOX | 1.096 | 0.772-1.557 | 0.607 |
| - GEMCITABINE | 1.706 | 1.149-2.533 | 0.008 |
| **Evolutionary events** |  |  |  |
| Gemcitabine dose reduction | 1.123 | 0.800-1.543 | 0.211 |
| Platinum dose reduction | 1.162 | 0.912-1.915 | 0.156 |
| Platinum withdrawal | 1.409 | 1.038-1.911 | 0.028 |
| First line permanent interruption | 42.843 | 15.987-114.818 | <0.001 |
| Progressive disease | 16.279 | 8.500-31.179 | <0.001 |
| Ascites | 4.811 | 3.254-7.112 | <0.001 |
| Bilirubin > 3 mg/dl | 5.831 | 3.871-8.784 | <0.001 |
