# Supplementary material for: Pattern of progression of intrahepatic cholangiocarcinoma: Implications for second‐line clinical trials
Source: Liver Int. 2021 Dec 10;42(2):458–67. doi: 10.1111/liv.15117 (PMC9300150; doi:10.1111/liv.15117)
Supplement: Supplementary file 2 — Table S2 [file LIV-42-458-s002.docx]

**SUPPLEMENTARY TABLE 2.** Multivariate Cox analysis of postprogression survival in patients with radiologic tumor progression under gemcitabine-cisplatin and eligible for a second-line trial. This model included sensitivity to platinum instead of time to progression, as the two variables were co-linear.

| **Variable** |  |  |  |
| --- | --- | --- | --- |
|  | HR | 95% CI | P |
| PS>0 | 2.659 | 1.266-5.581 | 0.010 |
| Platinum sensitive (vs resistant/refractory) | 0.558 | 0.332-0.895 | 0.016 |
| NEH lesion | 2.532 | 1.314-4.880 | 0.006 |

HR: hazard ration; CI: confidence interval; PS: performance status; NEH: new extrahepatic.
